# Knockdown of MSI2 inhibits metastasis by interacting with caveolin-1 and inhibiting its ubiquitylation in human NF1-MPNST cells

**DOI:** 10.1038/s41419-020-2703-x

**Published:** 2020-06-30

**Authors:** Kang Yang, Jianwei Du, Dai Shi, Feng Ji, Yong Ji, Junbo Pan, Fei Lv, Yao Zhang, Jie Zhang

**Affiliations:** 1 Department of Orthopedics, Yangzhou University Affiliated Hospital, No. 368, Hanjiang middle Rd, 225000 Yangzhou, People’s Republic of China; 2 Department of Urology, Sir Run Run Shaw Hospital, No. 3, Qingchun east Rd, 310000 Hangzhou, People’s Republic of China

**Keywords:** Sarcoma, CNS cancer

## Abstract

Malignant peripheral nerve sheath tumours (MPNSTs) are highly aggressive Schwann cell-derived sarcomas, and they are either associated with neurofibromatosis type 1 (NF1) or sporadic. Our previous study found that high mobility group protein A2 (HMGA2) regulates NF1-MPNST growth through Musashi-2 (MSI2); however, whether MSI2 regulates MPNST metastasis and what the mechanism is remain unclear. Here, we demonstrated that the protein caveolin-1 (CAV1) directly interacts with MSI2 in human NF1-MPNST cells. Moreover, we discovered that knockdown of MSI2 induces CAV1 protein expression by inhibiting its ubiquitylation level in NF1-MPNSTs. In addition, CAV1 mediates the suppressive function of MSI2 in epithelial-mesenchymal transition, migration and invasion in vitro and metastasis in vivo. These results help to reveal the potential mechanisms of MSI2 as a target of antimetastatic treatment for human NF1-MPNST.

## Introduction

Malignant peripheral nerve sheath tumours (MPNSTs) are highly aggressive Schwann cell-derived sarcomas that are either associated with NF1 or sporadic^1^. NF1-MPNSTs are malignant tumours that are transformed from neurofibromas^2^. Once malignant transformation occurs, the patient’s prognosis is very poor, with death often resulting from lung metastasis^3^.

The Musashi family includes Musashi-1 (MSI1) and Musashi-2 (MSI2). Both have about 75% amino acid identity in the overall structure and belong to the RNA binding protein family^4^. MSI1 and MSI2 are highly expressed in various tumours such as glioma, breast cancer, pancreatic cancer, colon cancer, lung cancer, ovarian cancer and prostate cancer^5–10^. Whether MSI2 or MSI1, their expression levels in tumours are higher than those of corresponding normal tissues, and are related to tumour differentiation level, poor prognosis of patients, number of lymph node invasions and distant metastasis. For most solid tumours, the key to transforming from a localised tumour to an aggressive or metastatic tumour is the transformation of the cellular state, in which the epithelial characteristics and lateral connections between cells are lost and then replaced by the mesenchyme that supports cell migration. Epithelial-mesenchymal transition (EMT) is the direction of many studies, not only related to tumour grade, but also closely related to stem cell status and drug resistance. In a variety of tumours, knocking down MSI2 can inhibit invasion and EMT protein expression. The main mechanisms include the TGFβ signaling pathway, PDK–AKT–mTORC1 signaling pathway and WNT and Notch signaling pathways. Our previous study found that knockdown of HMGA2 inhibits NF1-MPNST growth through MSI2 and that MSI2 expression in NF1-MPNSTs is higher than that in neurofibromas. We also found that MSI2 can interact with Beclin1, an autophagy marker, inducing NF1-MPNST cell autophagy^11^. Whether MSI2 can regulate cell metastasis in NF1-MPNST remains unclear. Some molecular pathways that explain the function of MSI2 have been partially elucidated, and clear identification of MSI2 targets and partners still needs further exploration.

Caveolin-1 (CAV1), a member of the membrane-bound scaffolding Caveolin family^12^, is crucial for signal transduction and vesicular trafficking^13^. CAV1 is ubiquitously expressed and involved in multiple cellular processes, such as cell proliferation, adhesion, migration and invasion^14,15^.

In this study, we report for the first time a relationship between MSI2 and CAV1 in regulating NF1-MPNST metastasis. Our results showed that MSI2 can directly interact with CAV1 and that MSI2 knockdown increases CAV1 protein levels by inhibiting CAV1 ubiquitylation. In addition, CAV1-mediated cell EMT and metastasis is inhibited by knockdown of MSI2 in vitro, and the metastatic effect was also validated by in vivo experiments.

## Materials and methods

### Human microarray sample analysis

We collected microarray expression profiles of normal human nerves, neurofibromas and MPNSTs from the Gene Expression Omnibus (GEO) public database; the accession numbers are GSE41747 and GSE66743. Normalized values from these datasets were analysed to generate gene expression scores. The expression levels of Musashi-2 (MSI2) and caveolin-1 (CAV1) in nerves, neurofibromas and MPNSTs were plotted using SPSS 20.0. *P*-values <0.05 were considered to indicate statistically significant differences.

### Clinical specimens

Sixty-one paraffin-embedded tissue samples (including 25 NF1-MPNSTs and 36 sporadic MPNSTs) were acquired from the Department of Orthopedics, Yangzhou University Affiliated Hospital (Jiangsu, China). The clinical characteristics of these 61 paraffin-embedded samples are shown in Table 1. Clinical and histopathologic information was recorded through a retrospective review of patient records.

**Table 1:** Association between clinicopathologic characteristics and CAV1 expression.

			cav1 expression		
Clinicopathological variables		*N*	Positive	Negative	*p*-value
Sex	Male	29	13	16	0.263
	Female	32	18	14	
Age(year)	≥40	35	18	17	0.529
	<40	26	14	12	
Pathogenic site	Extremities	31	14	17	0.351
	Trunk	30	16	14	
Histopathological	NF1 MPNST	25	8	17	0.038*
	Sporadic MPNST	36	21	15	

*MPNST* malignant peripheral nerve sheath tumours.

*p < 0.05.

### Cell culture and reagents

The human MPNST cell lines sNF96.2 and sNF02.2 were purchased from ATCC (ATCC, Manassas, VA), while ST8814 and STS26T cells were kind gifts from Dr. Yang Jilong (Tianjin Medical University, China) and Dr. Nancy (Cincinnati Children’s Hospital Medical Center, USA). All cells were cultured in Dulbecco’s modified Eagle’s medium (Gibco) supplemented with 10% FBS, and they were maintained at 37 °C in a humidified atmosphere with 5% CO_2_.

The following antibodies were used in the experiments: anti-E-cad, anti-N-cad, anti-Vimentin and anti-GAPDH antibodies from Cell Signaling Technology (Beverly, MA, USA); anti-MSI2 and anti-CAV1 antibodies from Abcam (Cambridge, MA, USA),anti-ubiquitin (FK2) from Enzo Life Sciences(New York, NY, USA).

### Transfection

The lentiviral vectors pLKO.1-MSI2 (shMSI2), pLKO.1-CAV11 (shCAV1), pLKO.1-Scramble (shScr), pLVX-Puro-CAV1 (CAV1) and pLVX-Puro-Control (Ctr) were constructed and used for lentivirus production in HEK293T cells. The NF1-MPNST cell lines ST8814 and sNF96.2 were transfected with lentiviral vectors. Stable cells were selected by treatment with puromycin (1.5 μg/ml) for 4 weeks. All primers used in this study are listed in Supplemental Table 2.

### Western blotting (WB) analysis

Protein samples were prepared using RIPA lysis buffer [25 mmol/l Tris-HCl (pH 7.5), 150 mmol/l NaCl, 1 mmol/l EDTA, 1% Triton X-100] containing a protease inhibitor cocktail tablet (Roche Applied Science). Proteins were separated via SDS-PAGE and transferred to a nitrocellulose membrane. After blocking with Tris-buffered saline containing 5% skim milk and 0.1% Tween-20 for 1 h at room temperature, the membrane was incubated with a primary antibody at 4 °C overnight. The next day, the membrane was washed and incubated with a goat anti-mouse or a goat anti-rabbit secondary antibody (Boster) for 1 h at room temperature, and enhanced chemiluminescence was used to visualize the protein bands in a Bio-Rad ChemiDoc XRS Imaging System.

### Immunohistochemistry (IHC)

IHC was performed as previously described^11^. The number of cells exhibiting positive staining at the cell membrane and in the cytoplasm and nucleus was counted in at least 10 representative fields (×400 magnification). Immunostaining was assessed by two independent pathologists blinded to clinical characteristics and outcomes.

### Quantitative real-time polymerase chain reaction (qRT-PCR)

Total RNA was extracted using TRIzol (Invitrogen), and reverse transcription was performed using the Advantage RT-for-PCR Kit (Takara Bio) according to the manufacturer’s instructions. For real-time PCR analysis, dsDNA was amplified using the SYBR Green PCR Kit (Takara Bio). The cycling parameters were as follows: 95 °C for 1 min, followed by 45 cycles of 95 °C for 10 s and 55–60 °C for 30 s. A melting curve analysis was then performed. Cycle threshold (Ct) values were measured during the exponential amplification phase, and amplification plots were analysed using CFX96 software (Bio-Rad). Expression levels were normalized to the fold change in corresponding control cells, which was defined as 1.0. All reactions were performed in triplicate.

### RNA immunoprecipitation

RNA immunoprecipitation (RNA-IP) was performed using Magna RIP RNA Binding Protein Immunoprecipitation Kit (Millipore, Billerica, MA) according with manufacturer’s instructions. In brief, cells were washed with cold phosphate-buffered saline and lysed with RIPA lysis buffer provided in the kit. Next, 5 μg of anti-MSI2 or anti-IgG control antibody was incubated with magnetic beads, and used to immunoprecipitate endogenous MSI2-RNA complexes. After the immunoprecipitated complexes were washed, they were treated with proteinase K. RNA extraction was performed by the phenolchloroform method, and purified RNA was used for qRT-PCR to check RNA binding with MSI2 protein. Results are presented relative to IgG immunoprecipitation, set as 1.

### Transwell and matrigel invasion assays

A total of 5 × 10^4^ cells was seeded in media without FBS in the top chamber of non-coated (3422, Corning) or matrigel-coated (354480, Corning).Transwell plates with membranes containing 8.0-μm pores.Medium with 10% FBS was placed in the bottom chamber. Twenty-four hours after seeding, noninvasive cells in the top chamber were removed with a cotton swab, and the cells on the lower surface of the membrane were fixed, stained with crystal violet and photographed at ×200 magnification using an Olympus BX51 microscope. Photographs of three random fields from three wells of each experiment were recorded, and the number of cells was counted.

### Co-immunoprecipitation (IP) and mass spectrometry analysis

Immunoprecipitation was performed as previously described^11^. Samples were separated by 10% SDS-PAGE, which was followed by Coomassie blue staining. The gel was cut into four segments and submitted to shotgun proteomics analyses using an EASY-nLCTM 1200 UHPLC system (Thermo Fisher) coupled to an Orbitrap Q Exactive HF-X mass spectrometer (Thermo Fisher), which operated in data-dependent acquisition (DDA) mode. The resulting spectra from each fraction were searched separately against the Homo_sapiens_UniProt (169389 sequences) database, and the results are shown in Supplemental Table 1.

### Wound-healing assay

Cells were seeded in 6-well plates and cultured for 24 h. When the confluency reached 80%, a 200-μl pipette tip was used to make a straight artificial wound. Image acquisition was performed using a phase-contrast microscope (Leica, Leica Microsystems).

### GST pull-down assay

A GST-vector or GST-MSI2 fusion proteins were purified from bacteria and were immobilized on GST beads (GE Healthcare); then, they were incubated with lysates prepared from HEK293T cells transiently transfected with His-tagged CAV1 for 2 h at 4 °C. The samples were washed five times and analysed by western blotting.

### Caveolin-1 ubiquitylation assay

Cells were treated with Mg132 (5 μg/ml) for 6 h to inhibit proteasome activity, and then they were lysed using SDS-free RIPA buffer, immunoprecipitated with an anti-Caveolin-1 antibody, and incubated with protein G plus agarose. The samples were then subjected to SDS-PAGE to detect Caveolin-1 and ubiquitin (FK2, Enzo Life Sciences, New York, NY, USA).

### Orthotopic mouse model and in vivo luciferase imaging

NSG female mice (6 to 8 weeks old) were obtained from SPF Biotechology Co., Ltd. (Beijing, China). A total of 2 × 10^6^ cells (ST8814) were injected into the tail vein (*n* = 4 per group). After 8 weeks, mice were injected intraperitoneally with D-luciferin (Caliper Life Sciences) and allowed to move freely for 10 min to promote substrate absorption. After being anaesthetized, the mice were subjected to whole-body live imaging using an IVIS Imaging System (Caliper Life Sciences). The mice were sacrificed immediately thereafter, and the lungs were fixed in formalin, embedded in paraffin, sectioned, and stained with haematoxylin and eosin (H&E) for analysis.

### Statistical analysis

Data represent the mean ± SD. All statistical analyses were conducted using the SPSS 20.0 software package. Statistical tests were one-sided or two-sided, and differences between two groups were assessed using Student’s *t*-tests, while ANOVA was used to compare multiple groups. Overall survival curves were estimated using the Kaplan–Meier method, and differences in survival were evaluated using the log-rank test. *P* < 0.05 was considered to indicate a statistically significant difference.

## Results

### Knockdown of MSI2 suppresses migration and invasion of NF1-MPNST cells

**Fig. 1:**
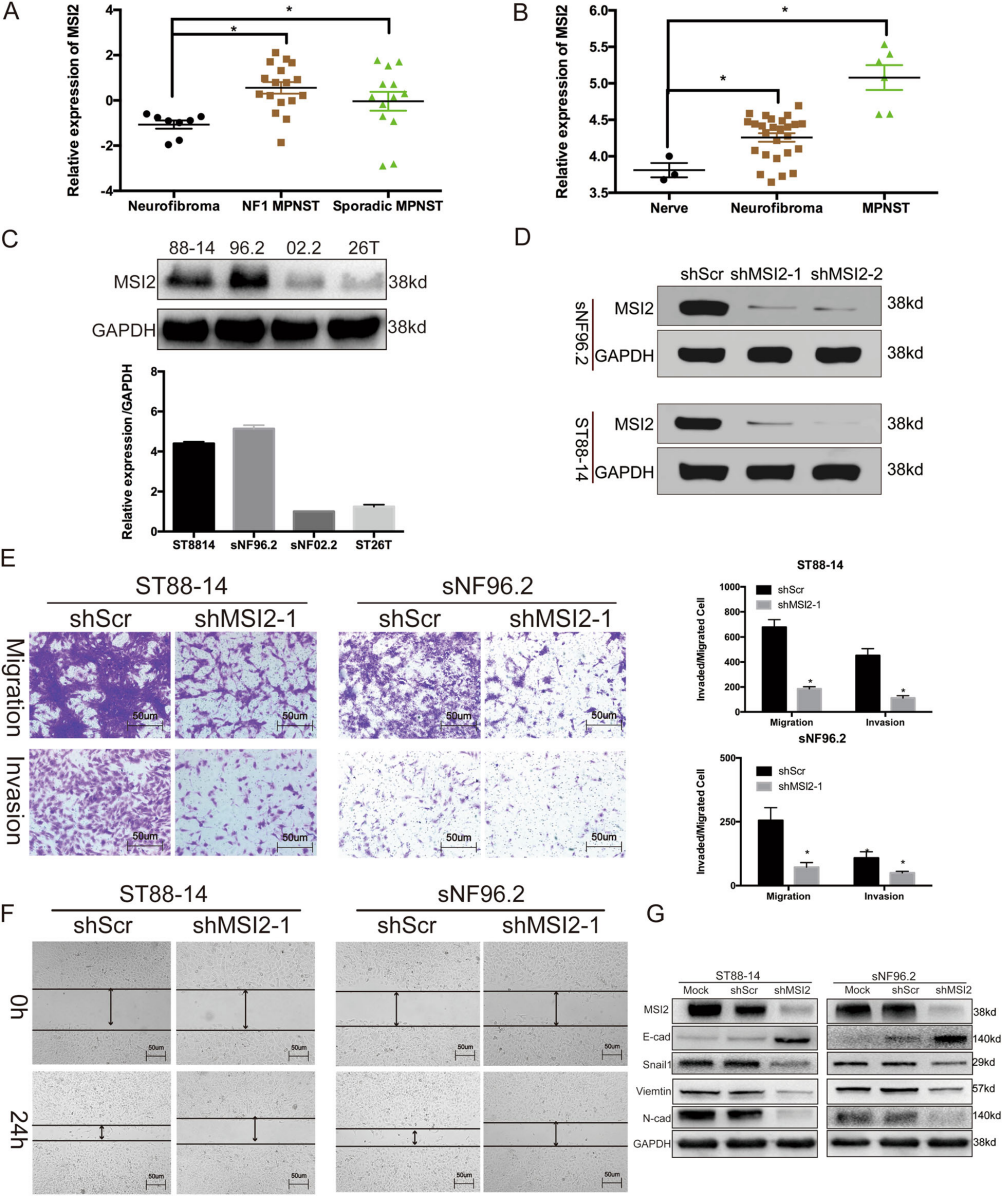
Knockdown of MSI2 suppresses migration and invasion of NF1-MPNSTs. **a** Average expression of MSI2 in MPNSTs (*n* = 30) and neurofibromas (*n* = 8) in the Kolberg cohort. **b** Average expression of MSI2 in MPNSTs (*n* = 6) and neurofibromas (*n* = 26) relative to that in normal nerves (*n* = 3) in the Jessen cohort. **c** MSI2 expression was higher in the NF1-deficient MPNST cell lines (ST8814 and sNF96.2) than in the NF1-expressing cell lines (sNF02.2 and STS26T). GAPDH was used as a control. The relative MSI2 protein expression level is shown as a percentage of GAPDH expression. **d** Knocking down MSI2 and verify knockdown efficiency through western Blot. **e** shMSI2 treatment significantly suppresses the migration (up) and invasion (down) of NF1-MPNST cells, as determined by transwell assays. Representative photos of stained cells are shown with the original magnification of ×100; the scale bar represents 50 μm.(*P < 0.05). **f** Wound-healing assays of ST8814 and sNF96.2 cells. Cells were transfected with shMSI2, the migration of ST8814 and sNF96.2 cells was significantly decreased. The representative images were shown at magnification of ×50, the scale bar represents 50 μm. **g** Immunoblot results of molecular markers of EMT after shMSI2 treatment. The results are typical of three to five experiments.

Our previous study found that MSI2 knockdown inhibits cell proliferation in NF1-MPNSTs^11^, so we wanted to determine whether MSI2 could regulate NF1-MPNST cell migration and invasion. We first determined whether MSI2 expression was significantly higher in NF1-MPNSTs than it was in neurofibromas through analysis of two MPNST patient cohorts, Jessen_cohort (GEO: GSE41747) and Kolberg_cohort (GEO: GSE66743). As shown in Fig. 1a and b, the MSI2 score in NF1-MPNSTs was significantly higher than the score in neurofibromas. We also analysed four MPNST cell lines and found that ST8814 and sNF96.2 cell lines showed higher levels of MSI2 protein than sNF02.2 and STS26T cells did (Fig. 1c). We then chose ST8814 and sNF96.2 as our target cell lines, which were also NF1-MPNST(NF1^−/−^) cell lines^11^.

We established ST8814 and sNF96.2 cell lines that constitutively and stably inhibited MSI2 expression (Fig. 1d). As shown in the transwell assay (Fig. 1e), knockdown of MSI2 in ST8814 and sNF96.2 cells resulted in an approximately 63–72% reduction of migratory capacity and an approximately 54–73% reduction of invasive capacity. Our previous studies found that knocking down MSI2 inhibited the growth of cells. In order to rule out the effect of cell proliferation, we did wound-healing assay and found that cells in the shMSI2 group migrated significantly slower.

More importantly, to further determine the function of MSI2 in the regulation of EMT, the key protein Snail 1,E-cad, N-cad and Vimentin were also examined. The results indicated that Snail 1, E-cad, N-cad and Vimentin were involved in MSI2-mediated EMT modulation. Specifically, shMSI2 transfection resulted in increased expression of E-cad while a reduction in Snail 1, N-cad and Vimentin levels was observed in both cell lines.

The results confirmed that MSI2 knockdown inhibits migration and invasion in NF1-MPNST cells and demonstrated that MSI2 can regulate EMT.

### Knockdown of MSI2 inhibits Caveolin-1 ubiquitylation and degradation in vitro

**Fig. 2:**
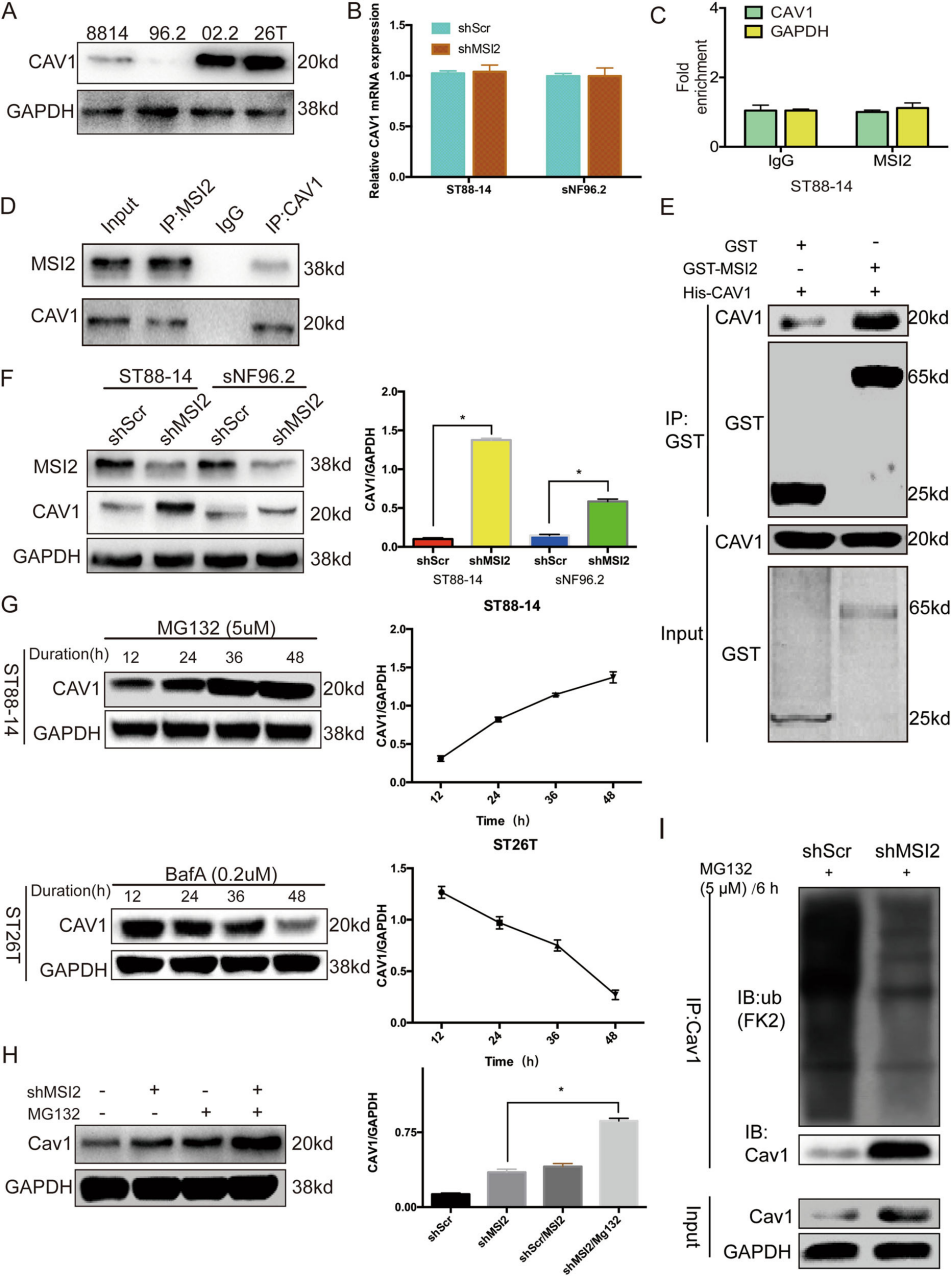
Knockdown of MSI2 inhibits caveolin-1 ubiquitylation and degradation in vitro. **a** CAV1 expression is lower in the NF1-deficient MPNST cell lines (ST8814 and sNF96.2) than in the NF1-expressing cell lines (sNF02.2 and STS26T). **b** qRT-PCR analysis was performed to detect CAV1 mRNA expression in control-, shMSI2-transfected NF1-MPNST cells. Bars represent the SEM. **P < 0.05 by Student’s *t*-tests. **c** RNA-immunoprecipitation in ST8814 cells using an anti-IgG control or anti-MSI-2 antibody. CAV1 or GAPDH mRNA abundance in immuneprecipitated fraction was determined by qRT-PCR. Results are presented relative to IgG immunoprecipitation, set as 1. **d** IP and WB data verify interactions between MSI2 and CAV1 in ST8814 cells. **e** Beads coated with GST or GST-MSI2 fusion proteins were incubated with the His-CAV1 protein overnight. The GST pull-down was immunoblotted with the indicated antibodies. **f** Knockdown of MSI2 significantly elevates CAV1 protein levels, GAPDH was used as a control. The relative CAV1 protein expression level is shown as a percentage of GAPDH expression. **g** Accumulation of CAV1 is shown after treatment with 5 μM MG132 in ST8814 cell, an inhibitor of the ubiquitin-proteasome system, and 0.2 μM lysosomal inhibitor Bafilomycin A1 (BafA) in ST26T; data were analysed at the indicated time points by Western blot. GAPDH was used as a control. The relative CAV1 protein expression level is shown as a percentage of GAPDH expression. **h** CAV1 protein levels are shown from shScr and shMSI2-transfected ST8814 cells treated with or without MG132 (10 μM) for 48 h. GAPDH was used as a control. The relative CAV1 protein expression level is shown as a percentage of GAPDH expression. **i** CAV1 was immunoprecipitated using an anti-CAV1 antibody from control cells and cells transfected with shMSI2. Western blots with an anti-ubiquitin (FK2) antibody reveal that the CAV1 ubiquitination level in immunoprecipitates prepared from shMSI2 cells was much lower than it was in control cells.

To investigate which proteins MSI2 interact with, we used co-immunoprecipitation (IP) and mass spectrometry analysis. As shown in Supplemental Table 1, of the proteins that MSI2 can interact with, Caveolin-1(CAV1) has one of the top mass spectrometry scores. We then measured the protein level of CAV1 in four MPNST cell lines and found that MSI2 expression is the opposite of CAV1 protein expression. (Fig. 2a). MSI2 is an RNA binding protein, whether it regulates the transcription level of CAV1 is not clear, so we knocked down MSI2 and found that it does not affect the CAV1 transcription level through qRT-PCR experiments(Fig. 2b). We also found that MSI2 could not bind to CAV1 mRNA through RNA-immunoprecipitation experiments (Fig. 2c). To determine whether MSI2 protein interacts with CAV1 protein in cell lines, we performed co-immunoprecipitation experiments in ST8814 cell using the MSI2 antibody, and we found that CAV1 could be pulled down; further, the CAV1 antibody was used to pull down MSI2 (Fig. 2d), confirming the interaction between MSI2 and CAV1 in NF1-MPNST cells. To verify that MSI2 can directly interact with CAV1 we performed GST pull-down of GST-tagged MSI2 with His-tagged CAV1, and the pulled down GST-MSI2 and His-tagged full-length CAV1 fractions were analysed by SDS-PAGE and Western blotting (Fig. 2e). The results confirmed that MSI2 can directly interact with CAV1. We later found that CAV1 protein expression was significantly elevated in cells treated with shMSI2 (Fig. 2f). Because MSI2 physically interacts with CAV1 and reduces CAV1 protein expression in NF1-MPNST cell lines, we then investigated whether CAV1 degradation could be modulated by MSI2. A previous study has found that NDRG1 regulates the CAV1 protein level through ubiquitylation^16^, and whether MSI2 also regulates CAV1 through ubiquitylation needed to be determined.

The Caveolin family degrades proteins in two main ways, the proteasome and lysosome pathways^17,18^, so we needed to determine which pathway was used in CAV1 degradation. We treated cells with the lysosomal inhibitor Bafilomycin A1 (BafA) in CAV1 high expression cell line ST26T and found that it had no effect on CAV1 degradation, but it did increase Cav1 degradation. While the proteasomal inhibitor MG132 markedly increased CAV1 protein levels in a time-dependent manner (Fig. 2g) in CAV1 low expression cell line ST8814, indicating that CAV1 is mainly degraded via the proteasomal pathway in NF1-MPNST cells. We then chose to use MG132 in further investigation. As shown in Fig. 2h, MG132 significantly increased the expression of CAV1 protein in shMSI2 cells compared to that in shMSI2 cells alone. These results indicate that MSI2 can regulate CAV1 protein expression by promoting proteasomal degradation.

In proteasome-mediated protein degradation, proteins are usually conjugated via isopeptide linkages to form polyubiquitinated proteins^19,20^. Therefore, inhibition of the proteasome pathway, can lead to the accumulation of ubiquitinated proteins. To test whether MSI2 plays a role in ubiquitination of the CAV1 protein, cells with MSI2 knocked down were used for immunoprecipitation assays. The results showed that the anti-ubiquitin CAV1 antibody (FK2) showed multiple corresponding bands (Fig. 2i), demonstrating that shMSI2 inhibits the ubiquitination of CAV1 in ST8814 cells. All data indicate that the increase in CAV1 after MSI2 knockdown is due to CAV1 ubiquitination and subsequent proteasomal degradation.

### CAV1 is negatively correlated with MSI2 and is associated with patient prognosis and lung metastasis

**Fig. 3:**
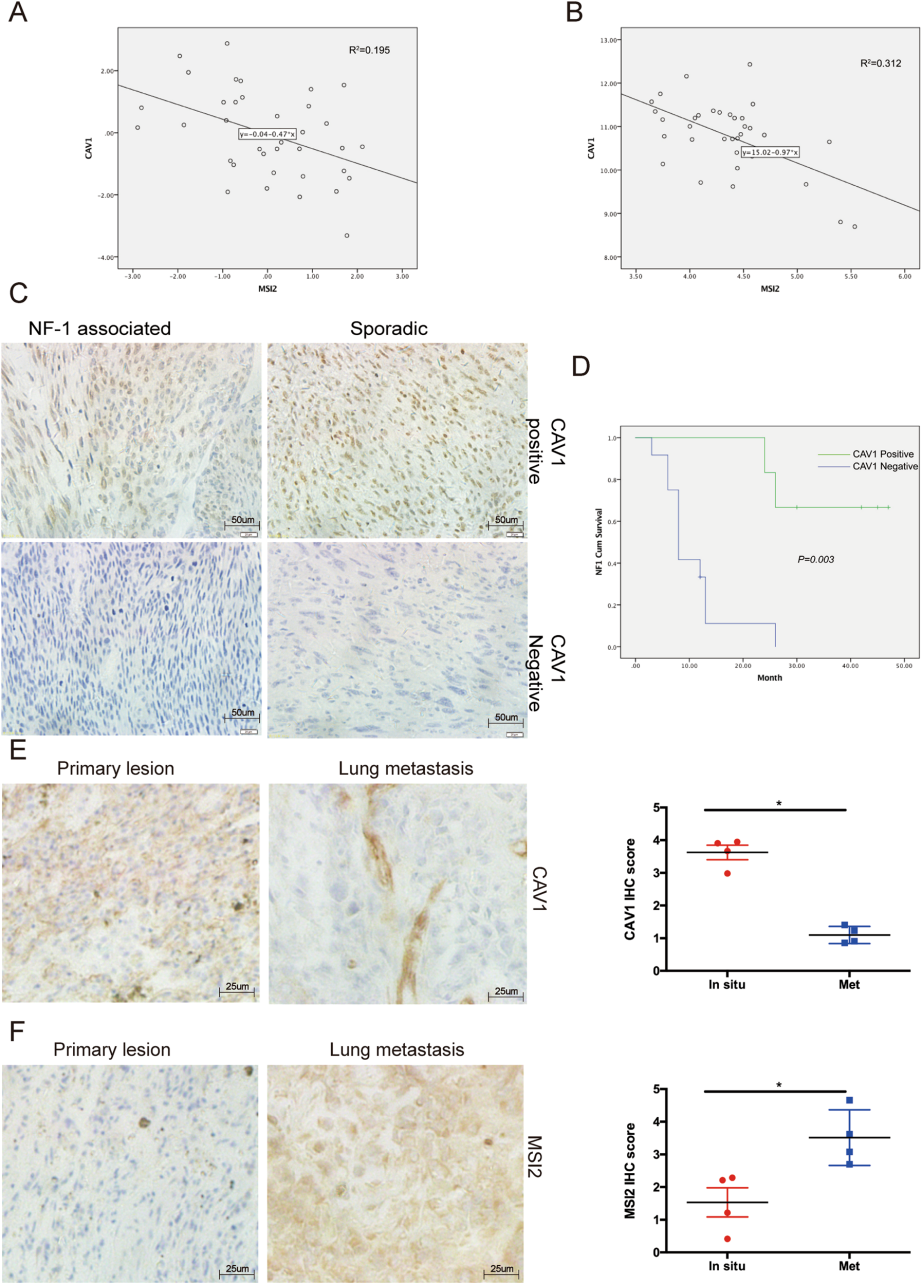
CAV1 is negatively correlated with MSI2 and is associated with patient prognosis and lung metastasis. **a**, **b** Correlation analysis between MSI2 and CAV1 expression levels in the the Kolberg cohort and Jessen cohort. **c** IHC results show positive and negative CAV1 staining in NF1-MPNST samples and sporadic MPNST samples. Representative photos are shown with the original magnification of ×100; the scale bar represents 50 μm. **d** Overall survival of NF1-MPNST patients with positive CAV1 staining. **e** Representative images of CAV1 immunostaining of paired specimens of NF1-MPNST lung metastases and primary lesions are shown at ×200 magnification. the scale bar represents 50 μm. **f** Representative images of MSI2 immunostaining of paired specimens of NF1-MPNST lung metastases and primary lesions are shown at ×200 magnification. the scale bar represents 50 μm.

Because MSI2 can regulate CAV1 expression through ubiquitylation, we assessed whether these two genes are associated with patient prognosis. We first detected the correlation between MSI2 and CAV1 expression in two GEO databases and found that the CAV1 score is negatively correlated with the MSI2 score in two MPNST patient cohorts, Jessen_cohort (GEO: GSE41747) and Kolberg_cohort (GEO: GSE66743) (Fig. 3a, b). We then examined the expression rate of CAV1 in NF1 and sporadic MPNST patient samples by immunohistochemistry (Fig. 3c). The results revealed that the CAV1-positive staining rate in NF1-MPNST (8/25) is lower than it is in sporadic MPNST patients (21/36) (Table 1). Because the prognosis for patients with NF1-MPNST is very low, it is important to improve the treatment options for these tumours. Whether CAV1 is related to patient prognosis is not clear. Therefore, we explored the relationship between the positive staining of CAV1 and the survival rate of patients through Kaplan–Meier survival analysis. As shown in Fig. 3d, patients with positive staining for CAV1 had a better prognosis (*p* = 0.003).

Then, we wanted to understand the relationship between MSI2, CAV1 and lung metastasis in patients with NF1-MPNST. We used immunohistochemistry to examine the positive staining rate of MSI2 and CAV1 in primary tumours and lung metastases. The results showed that in patients with lung metastasis, the MSI2 IHC score in the primary tumour was lower than it was in the lung metastases. In contrast, the CAV1 IHC score in the primary tumour was significantly higher than it was in the lung metastases (Fig. 3e, f).

These results indicate that CAV1 was negatively correlated with MSI2 expression. At the same time, patients with positive CAV1 staining have a better prognosis, and CAV1 is associated with lung metastasis in NF1-MPNSTs.

### Knockdown of MSI2 suppresses migration, invasion and EMT through CAV1

It has been reported that CAV1 acts not only as a tumour suppressor but also as a promoter of metastasis^21–23^. Regarding the tumour suppressor function of CAV1, its expression is inhibited in several human tumours, including lung, breast cancer, ovarian cancer and osteosarcoma^23–25^. Conversely, it has been reported that the presence of CAV1 is also associated with increased metastasis in prostate cancer^26^; therefore, whether CAV1 plays a role in preventing or promoting tumour progression appears to depend on the tumour type. Given that functional fluctuations in CAV1 are thought to be involved in the regulation of their interacting partners, the effect of MSI2 on cell migration and invasion may be mediated through downregulation of CAV1.

**Fig. 4:**
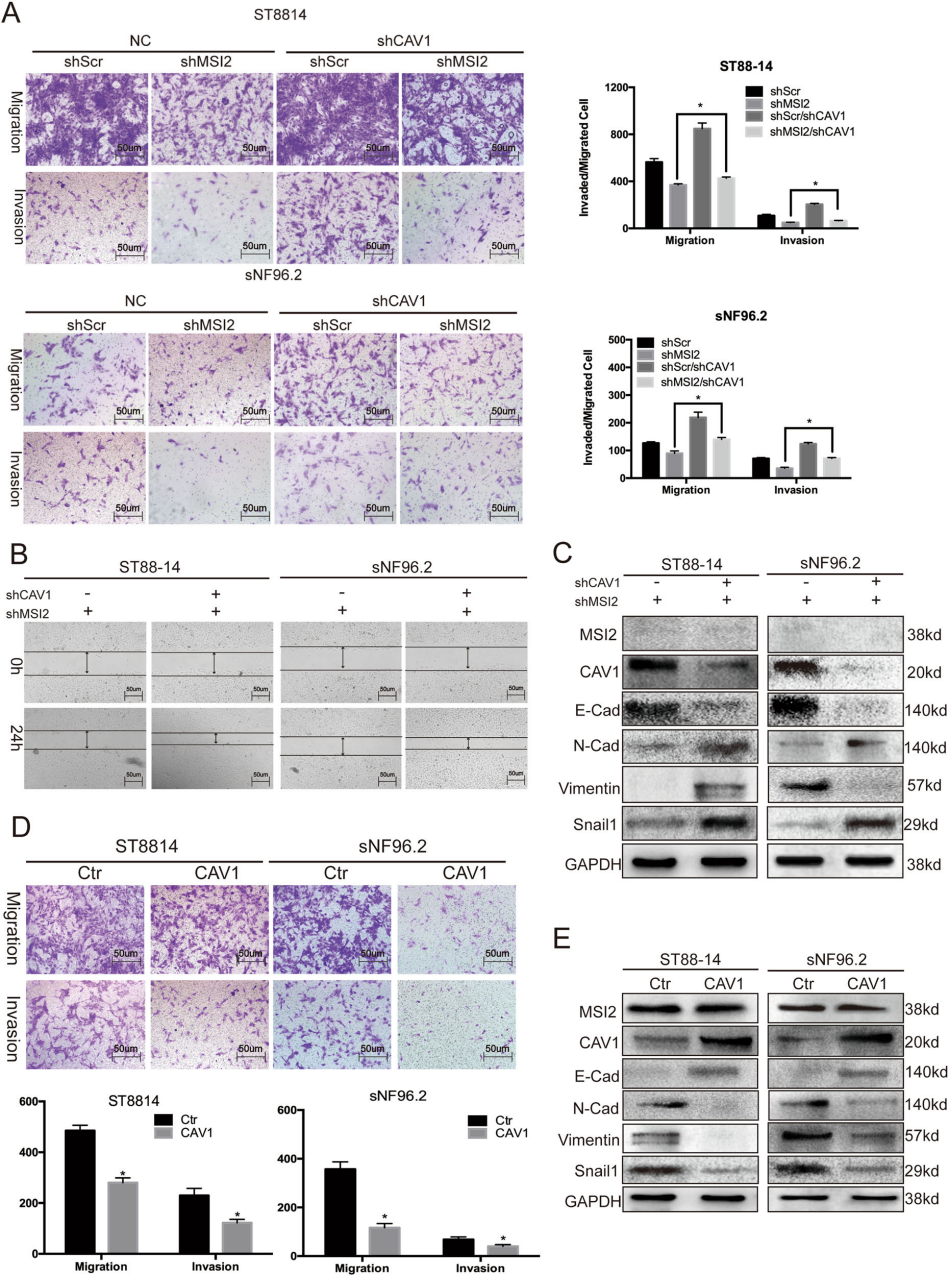
Knockdown of MSI2 suppresses migration, invasion and EMT through CAV1. **a** Transwell assays: cells were seeded in the upper chamber of the insert, and the migrated/invaded cells were examined after 24–48 h. Data represent the means ± s.d. from three independent experiments. Representative photos of stained cells are shown with the original magnification of ×100; the scale bar represents 50 μm. **b** Wound-healing assays of ST8814 and sNF96.2 cells. Cells were transfected with shMSI2,shMSI2/shCAV1, the migration of shMSI2/shCAV1 cells was significantly reversed. The representative images were shown at magnification of ×50. the scale bar represents 50 μm. **c** Immunoblot results of molecular markers of EMT after shMSI2 and/or shCAV1 treatment. The results are typical of three to five experiments. **d** Transwell assays were performed as follows: cells were seeded in the upper chamber of the insert, and the migrated/invaded cells were examined after 24–48 h. Data represent the means ± s.d. from three independent experiments. Representative photos of stained cells are shown with the original magnification of ×100; the scale bar represents 50 μm. **e** Immunoblot results of molecular markers of EMT after overexpression of CAV1. The results are typical of three to five experiments.

In transwell experiments, shMSI2 alone led to a significant reduction in migratory/invasive capacity. Notably, in the shMSI2/shCAV1 groups, the reduced migration and invasion capacity caused by silencing of MSI2 was completely abolished (Fig. 4a, b). We also tested EMT-related proteins and wanted to determine whether MSI2 could affect the EMT process through CAV1. In cells treated with shMSI2, the E-cad protein level was elevated, but the N-cad, Vimentin and Snail 1 protein levels were decreased; in shMSI2/shCAV1 cells, the protein levels were reversed (Fig. 4c).

On the other hand, overexpression of CAV1 alone significantly inhibited the migration and invasion ability of NF1-MPNST cells (Fig. 4d). At the same time, we overexpressed CAV1 alone in cells and found that CAV1 does not significantly affect the protein level of MSI2, but it can significantly inhibit the expression of EMT-related proteins (Fig. 4e).

The above results show that MSI2 knockdown suppresses migration, invasion and EMT through CAV1.

### Knockdown of MSI2 inhibits NF1-MPNST lung metastasis through CAV1 in vivo

**Fig. 5:**
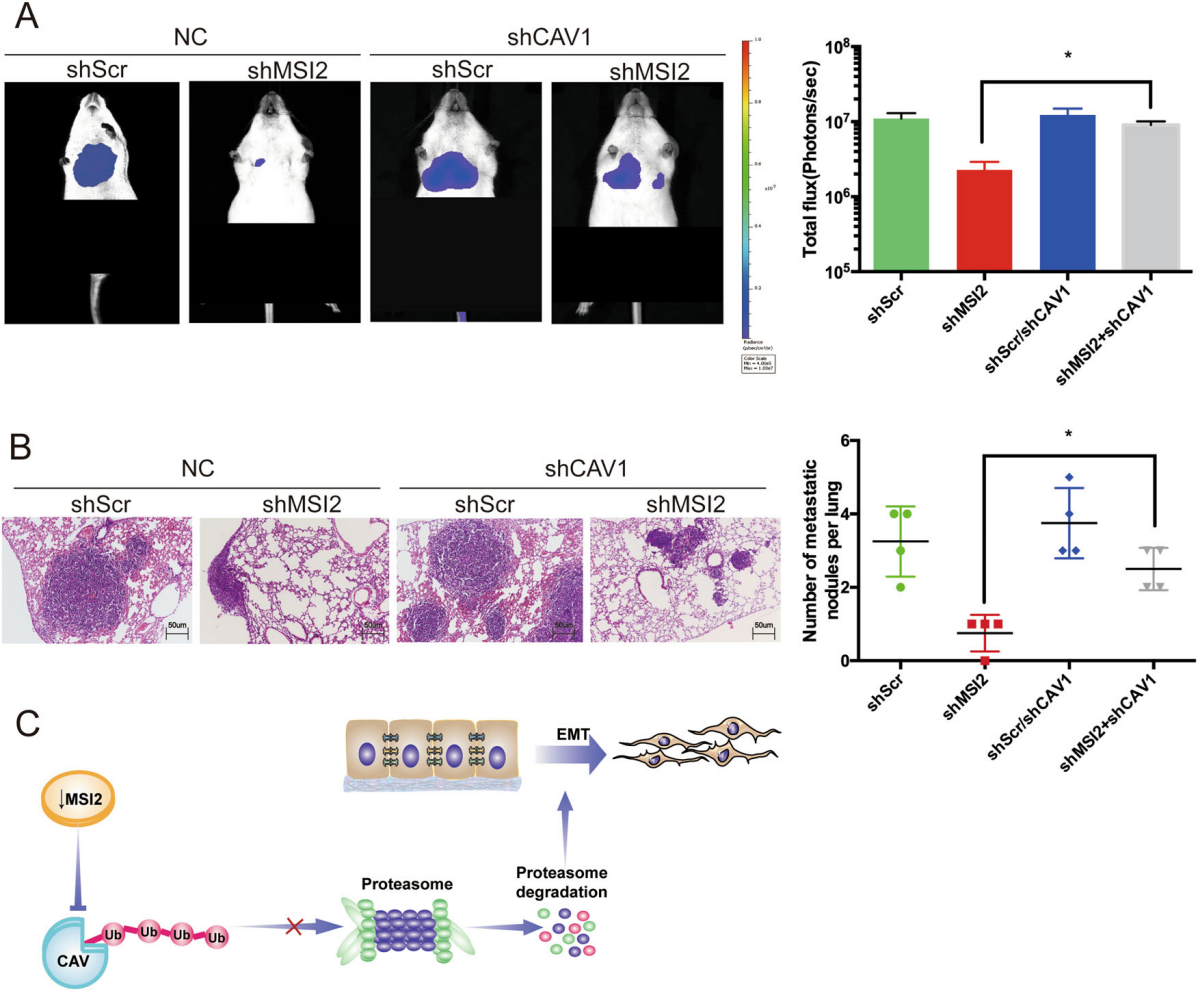
Knockdown of MSI2 inhibits NF1-MPNST lung metastasis through CAV1 in vivo. **a** Mice were imaged at 4 weeks after injection using an IVIS Imaging System. Three representative mice were imaged, and the colour scale depicts the photon fluxes emitted from the tumour cells. Monitoring of lung metastasis occurred via assessment of bioluminescence signals that were quantified using Living Image software, and the histograms represent the mean ± s.d. total fiux (photons/sec). *P < 0.05, relative to the respective control cells. **b** Metastases is visualized in haematoxylin and eosin (H&E)-stained lung sections; magnification: ×100 (*n* = 4 per group). Metastases were counted on five lobes of the lung in all animal groups. The results were collected and analysed from four nude mice per group, and the histograms represent the mean ± s.d. number of metastatic nodules. *P < 0.05, relative to the respective control cells. Representative photos are shown with the original magnification of ×100; the scale bar represents 50 μm. **c** Schematic representation of MSI2/CAV1-signalling-pathway-induced NF1-MPNST metastasis.

To verify the in vitro findings, we used an in vivo xenograft model. GFP-luciferase-labelled ST8814 cells with shMSI2 and shCAV1, and the appropriate control cells were injected into the tail veins of NSG mice (*n* = 4 per group). Luciferase photon fluxes were monitored for metastasis each week. Compared with the control group, knockdown of MSI2 significantly inhibited the lung fluorescence value of the animal model after 4 weeks, while the lung fluorescence value was significantly restored in the shMSI2/shCAV1 group (Fig. 5a). Haematoxylin and eosin (HE) staining showed that fewer lung metastatic nodes were detected in the group treated with shMSI2 compared to the control group, but the node number was restored in the shMSI2/shCAV1 group (Fig. 5b).

## Discussion

MPNSTs are highly malignant tumours; some MPNSTs are associated with the NF1 gene, and these tumours are mainly caused by the malignant transformation of neurofibromas^27^. In our previous study, we found that HMGA2 can regulate NF1-MPNST growth via MSI2^11^. Musashi-2 (MSI2) is an RNA binding protein, and several studies have implicated MSI2 in translational regulation that contributes to a variety of cancers^28,29^. MSI2 can regulate tumour progression through PTEN, Smad3, TGF-β, LRIG1 and others^8,28–30^. However, little is known about the mechanism of MSI2 in NF1-MPNST progression. Our previous study found that MSI2 can regulate NF1-MPNST growth through autophagy by interacting with Beclin1 (BECN1). Whether MSI2 regulates NF1-MPNST metastasis and what its mechanism of regulation is remains unclear. Here, we found that MSI2 can also regulate NF1-MPNST invasion through CAV1.

CAV1 plays a dual role in tumour progression, and it can inhibit or induce tumour progression depending on the tumour type. For example, it can inhibit osteosarcoma growth and invasion while inducing growth in CRC cells^16,23^. No study has found the function of CAV1 in NF1-MPNST progression, so in this study, we focused on the function and mechanism of CAV1 in regulating NF1-MPNST cells. First, through co-immunoprecipitation (IP) and mass spectrometry analysis, we found that MSI2 protein can interact with CAV1 protein.While MSI2 is an RNA binding protein and we found that MSI2 did not bind with CAV1 mRNA and did not affect CAV1 transcription level.Previous study found that CAV1 can be regulated through ubiquitylation to induce or inhibit tumour cell activity. Hence, suspected that MSI2 regulates CAV1 expression through ubiquitylation, and the results confirmed this hypothesis.

There is a negative correlation between MSI2 and CAV1 expression in the two sets of MPNST GEO data. According to clinical samples, patients with negative CAV1 staining have a worse prognosis. We also found that in patients with NF1-MPNST with lung metastasis, MSI2 was highly expressed in comparison to primary tumours, where CAV1 expression was the opposite; CAV1 expression was low in lung metastases and high in primary tumours.

In an in vitro experiment, MSI2 knockdown significantly inhibited the number of migrating and invading NF1-MPNST cells, while shMSI2/shCAV1 significantly reversed the number of migrating and invading cells; further, EMT-related protein levels were also reversed. On the other hand, overexpression of CAV1 alone did not affect MSI2 protein levels, but it did significantly inhibit the migration and invasion of NF1-MPNST cells and reduce the levels of EMT-related marker proteins.

Using in vivo experiments, we also demonstrated that knockdown of MSI2 inhibits cell lung metastasis, while treatment with shMSI2 and shCAV1 reverses lung metastasis. However, there are still some shortcomings in this study: NF1-MPNSTs are a kind of malignant transformation that originate from neurofibromas. On the one hand, we need to construct a transgenic model to determine whether the overexpression of MSI2 can induce an animal model of NF1-MPNST. Then, the model would be treated with MSI2 as a target. On the other hand, there are sometimes neurofibromas and MPNST regions in the same tumour area of NF1-MPNST. Further work could use single-cell sequencing to distinguish the neurofibroma area and the MPNST area from the same patient to understand the transformation of neurofibroma into MPNST.

## Conclusion

This present study shows that knockdown of MSI2 interacts with CAV1 and induces CAV1 protein expression by inhibiting CAV1 ubiquitylation, which modulates EMT in NF1-MPNST cells. These results help to explain the potential mechanisms of MSI2, which provides potential antimetastatic treatments for human NF1-MPNST(Fig. 5c).

## Supplementary information

Supplemental Table 1

Supplemental Table 2

## Data Availability

All data generated or analyzed during this study are included in this published article.
