# Correction: Knockdown of MSI2 inhibits metastasis by interacting with caveolin-1 and inhibiting its ubiquitylation in human NF1-MPNST cells

**DOI:** 10.1038/s41419-020-02816-z

**Published:** 2020-07-31

**Authors:** Kang Yang, Jianwei Du, Dai Shi, Feng Ji, Yong Ji, Junbo Pan, Fei Lv, Yao Zhang, Jie Zhang

**Affiliations:** 1 Department of Orthopedics, Affiliated Hospital of Yangzhou University, Yangzhou University, No. 368, Hanjiang Middle Road, 225000 Yangzhou, People’s Republic of China; 2 Department of Urology, Sir Run Run Shaw Hospital, No. 3, Qingchun east Rd, 310000 Hangzhou, People’s Republic of China

**Keywords:** Sarcoma, CNS cancer

Correction to: *Cell Death & Disease*

10.1038/s41419-020-2703-x published online 30 June 2020

The original version of this Article contained an error in the author affiliations.

Affiliation 1 incorrectly read ‘Department of Orthopedics, Yangzhou University Affiliated Hospital, No. 368, Hanjiang middle Rd, 225000 Yangzhou, People’s Republic of China’, whereas it should have read ‘Department of Orthopedics, Affiliated Hospital of Yangzhou University, Yangzhou University, No. 368, Hanjiang Middle Road, 225000 Yangzhou, People’s Republic of China’.

This has now been corrected in both the PDF and HTML versions of the Article.

