# Correction: Knockdown of MSI2 inhibits metastasis by interacting with caveolin-1 and inhibiting its ubiquitylation in human NF1-MPNST cells

**DOI:** 10.1038/s41419-026-08903-x

**Published:** 2026-06-02

**Authors:** Kang Yang, Jianwei Du, Dai Shi, Feng Ji, Yong Ji, Junbo Pan, Fei Lv, Yao Zhang

**Affiliations:** Department of Orthopedics, Affiliated Hospital of Yangzhou University, Yangzhou University, No. 368, Hanjiang middle Rd, 225000 Yangzhou, People’s Republic of China

Correction to: *Cell Death & Disease* 10.1038/s41419-020-2703-x, published online 30 June 2020

The authors wish to correct the author list. Jie Zhang was included in error and has been removed. This change does not affect the results or conclusions of the paper.

